# Characterisation of the effects of the chemotherapeutic agent paclitaxel on neuropathic pain-related behaviour, anxiodepressive behaviour, cognition, and the endocannabinoid system in male and female rats

**DOI:** 10.3389/fphar.2024.1505980

**Published:** 2025-01-03

**Authors:** Chiara Di Marino, Álvaro Llorente-Berzal, Alba M. Diego, Ariadni Bella, Laura Boullon, Esther Berrocoso, Michelle Roche, David P. Finn

**Affiliations:** ^1^ Pharmacology and Therapeutics, School of Medicine, University of Galway, Galway, Ireland; ^2^ Galway Neuroscience Centre, University of Galway, Galway, Ireland; ^3^ Centre for Pain Research, University of Galway, Galway, Ireland; ^4^ Department of Neuroscience, Neuropsychopharmacology and Psychobiology Research Group, University of Cádiz, Cádiz, Spain; ^5^ Physiology, School of Medicine, University of Galway, Galway, Ireland; ^6^ Instituto de Investigación e Innovación Biomédica de Cádiz (INiBICA), Hospital Universitario Puerta del Mar, Cádiz, Spain; ^7^ Centro de Investigación Biomédica en Red de Salud Mental (CIBERSAM), Instituto de Salud Carlos III, Madrid, Spain

**Keywords:** paclitaxel, chemotherapy, neuropathic pain, endocannabinoids, behaviour

## Abstract

Paclitaxel (PTX) is a commonly used chemotherapeutic drug, however, one of its major adverse effects is chronic neuropathic pain, with the incidence being higher in women than in men. The neurobiological mechanisms behind this sex difference are still largely unclear, and the endocannabinoid system, which exhibits sexual dimorphism and plays a key role in pain regulation, is a promising area for further studies. The present study aimed to characterise pain-, cognition-, anxiety-, and depression-related behaviours in male and female rats following PTX administration, and associated alterations in the endocannabinoid system. After the induction of the model, pain-related behaviours were assessed using von Frey, Acetone Drop and Hargreaves’ tests, Novel Object Recognition and T-Maze Spontaneous Alternation tests were used for cognition-related behaviours, Elevated Plus Maze, Open Field, and Light Dark Box tests were used to assess anxiety-related behaviours, and Sucrose Preference, Sucrose Splash, and Forced Swim tests for depression-related behaviours. At each time point analysed, animals treated with PTX exhibited mechanical and cold hypersensitivity, with females displaying lower hind paw withdrawal thresholds to mechanical stimulation than males. No PTX-induced alterations in the other behavioural tests were detected. *Post-mortem* measurement of endocannabinoid and related *N*-acylethanolamine levels in spinal cord and discrete brain regions revealed a PTX-induced increase of 2-Arachidonoyl Glycerol (2-AG), *N*-Palmitoylethanolamine (PEA) and *N*-Oleoylethanolamine (OEA) levels in the amygdala of male and female animals, but not in the other areas. Collectively, these results suggest that PTX causes similar long-lasting hypersensitivity to mechanical and cold stimuli, but not heat, in rats of both sexes, effects accompanied by increases in amygdalar levels of endocannabinoids and *N*-acylethanolamines.

## 1 Introduction

Paclitaxel (PTX) is a chemotherapeutic agent first extracted in 1971 from the bark of the Pacific yew tree (*Taxus brevifolia)* (Picard and Castells, 2015). It belongs to the class of antineoplastic drugs known as taxanes, which interfere with the normal cycle of microtubule de- and re-polymerisation (Alqahtani et al., 2019). Specifically, PTX interacts with β-tubulin through the microtubule lattice, increasing microtubule stability and polymerisation, with consequent cell death (Klein and Lehmann, 2021).

Between 1992 and 1994, the US Food and Drug Administration approved PTX for several cancer types, including ovarian, breast, non-small cell lung, prostate cancer, Kaposi sarcoma, gastric cancer, oesophageal cancer, bladder cancer, and other carcinomas (Tkaczuk and Yared, 2012), either as a monotherapy or in combination with other chemotherapeutic compounds (Armstrong et al., 2006; Loesch et al., 2010). It is mostly prescribed to female patients, primarily due to its use in treating many female-specific cancers. However, a significant limit to PTX usage is its dose-limiting adverse side effects. These adverse effects include alopecia, nausea, vomiting, and general hypersensitivity (i.e., dyspnoea, urticaria, hypotension, and fever), in addition to peripheral neuropathy which is considered one of the primary adverse effects in patients treated with this drug (Walker, 1993). In fact, PTX induces peripheral neuropathy in up to 97% of all treated patients, and 60% of patients subsequently develop chronic neuropathy (Tanabe et al., 2013), with symptoms that normally include weakness, numbness, and pain, typically in the hands and feet.

Cognitive impairment, often referred as “chemo brain”, is another adverse effect that patients experience even after 6 months following cessation of chemotherapy (Ibrahim et al., 2021). Common symptoms of chemo brain include learning problems, selective attention and memory impairment (Lindner et al., 2014). There can also be a higher prevalence of depression or anxiety in patients receiving chemotherapy, including taxanes (Ibrahim et al., 2021).

Despite the progress made to characterise the molecular mechanisms and the effects of chemotherapeutic drugs, the role of biological sex is often ignored. Women who receive chemotherapy experience more severe side effects when compared to men (Choi et al., 2024; Yamamoto et al., 2008), and in this context, preclinical models can be useful to further elucidate the mechanisms underlying the adverse effects induced by these compounds. However, most of the studies published so far in chemotherapy-induced peripheral neuropathy (CIPN) models have used only male rodents (Currie et al., 2019), overlooking sex as a variable and potentially missing important factors for advancing personalised treatment.

First line treatments for CIPN (typically include antidepressants, such as serotonin and norepinephrine reuptake inhibitors, and anticonvulsants, like gabapentinoids (Quintão et al., 2019). However, both classes of drugs have exhibited a limited effectiveness for this purpose. For instance, patients treated with duloxetine appeared comparable to those treated with placebo in several clinical trials (Chow et al., 2023). Similarly, gabapentin demonstrated a lack of efficacy in alleviating CIPN symptoms associated with increased pain scores in paediatric patients (Anghelescu et al., 2020).

Cannabis-based medicines, including nabiximols (Sativex^®^) or cannabidiol, have been shown to reduce chemotherapy-evoked neuropathic pain (Lynch et al., 2014; Weiss et al., 2023). Further evidence is provided by studies performed with the aid of preclinical models: AM1710, a cannabilactone CB_2_ agonist, induces antinociception in both cisplatin- and PTX-induced neuropathic pain models in rats (Deng et al., 2012). JZL184 and URB597, both compounds able to increase the levels of endocannabinoids through catabolic enzyme inhibition, reversed PTX-induced mechanical hypersensitivity in mice (Curry et al., 2018; Slivicki et al., 2019). These findings suggest a role for the endocannabinoid system in CIPN that requires further evaluation.

The endocannabinoid system is a complex neuromodulatory system that comprises the cannabinoid receptors 1 and 2 (respectively, CB_1_ and CB_2_), which are G_i/o_-coupled receptors (GPCRs) negatively coupled to adenylate cyclase (Howlett, 2002; Munro et al., 1993), as well as their endogenous ligands, known as endocannabinoids, and the enzymes that either synthesise or degrade them. Other receptor targets for endocannabinoids include transient receptor potential vanilloid 1 (TRPV1), and peroxisome proliferator-activated receptors (PPAR) (Lago-Fernandez et al., 2021). CB_1_ is expressed in both the peripheral and central nervous system, where it is widely distributed in neocortex, cerebellum, basal ganglia and limbic regions on the axon terminals and pre-terminal axon segments (Herkenham et al., 1991). Conversely, CB_2_ is mainly expressed in the cells and tissues of the immune system and in activated microglia in the brain (Klegeris et al., 2003; Galiègue et al., 1995). The two best-characterised endocannabinoids are anandamide (AEA) (Devane et al., 1992) and 2-arachidonoyl glycerol (2-AG) (Sugiura et al., 1995). The *N*-acylethanolamines, *N*-Palmitoylethanolamine (PEA) and *N*-Oleoylethanolamine (OEA), are also substrates for the AEA-catabolising enzyme fatty acid amide hydrolase (FAAH) and can alter levels of AEA via substrate competition at FAAH (Cravatt et al., 1996). On the other hand, 2-AG is degraded into arachidonic acid and glycerol by the presynaptic enzyme monoacylglycerol lipase (MGL) (Dinh et al., 2002). Interestingly, numerous lines of evidence indicate that the endocannabinoid system exhibits sexual dimorphism in levels of endocannabinoids and related *N*-acylethanolamines or expression of endocannabinoid system components (Bradshaw et al., 2006; Rubino and Parolaro, 2011; Blanton et al., 2021). This is particularly interesting considering the observed sex differences in humans treated with chemotherapeutic compounds, highlighting the need for further investigation to clarify its role and clinical potential.

Therefore, the aim of the present study was to characterise the effects of PTX on neuropathic pain-related behaviour, anxiodepressive behaviour, and cognition in rats of both sexes, and to define associated biochemical and molecular changes induced in the endocannabinoid system.

## 2 Materials and methods

### 2.1 Animals

Forty adult Sprague-Dawley (SD) rats (Males 280–300 g, Females 200–220 g, 9–10 weeks of age) were purchased from Charles River UK (United Kingdom). Animals were pair-housed in cages (males together and females together) with water and food (14% Harlan Teklad 2014 Maintenance Diet, Envigo, Huntingdon, Cambridgeshire, United Kingdom) available *ad libitum*, and all animals housed in the same holding room. The holding room was at a constant temperature of 21°C ± 2°C under standard light conditions (12:12 h light: dark, lights on from 07.00 to 19.00 h). Every procedure was performed during the light phase by a female researcher.

The experimental procedures were approved by the Animal Care and Research Ethics Committee (ACREC), University of Galway, in accordance with the ARRIVE guidelines, under licence from the Health Products Regulatory Authority in the Republic of Ireland (HPRA) and in accordance with EU Directive 2010/63.

### 2.2 Establishment of PTX-induced neuropathic pain model

Paclitaxel (PTX; Taxol–Tocris by Biotechne, United Kingdom) was dissolved in ethanol (Sigma-Aldrich, Ireland):Kolliphore (Sigma-Aldrich, Ireland):saline (1:1:18) to a concentration of 2 mg/mL. PTX (2 mg/kg/day) or its vehicle (VEH) was administrated via intraperitoneal injections (1 mL/kg injection volume) on four alternative days (day 0, 2, 4, 6), with the first injection occurring 28 days after arrival of the rats into the animal facility. Male and female rats were pseudorandomly allocated to either the PTX or VEH (n = 10 rats per group) groups following baseline pain-related behaviour testing to ensure no significant between-group differences in baseline behaviour.

### 2.3 Behavioural procedures

**FIGURE 1:**
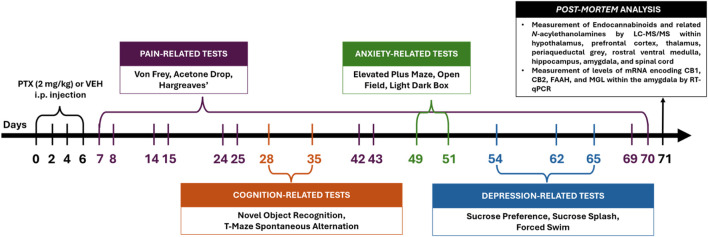
Schematic timeline of the experimental procedures (intraperitoneal injection indicated as i.p.).

All behavioural testing (Figure 1) was carried out by an experimenter blinded to PTX/VEH treatment.

#### 2.3.1 Pain-related behaviour

##### 2.3.1.1 Von Frey test

To measure mechanical hypersensitivity, the animals were placed on an elevated metal wire mesh, divided into six individual plexiglass compartments (14 × 20 × 25 cm) in which the light levels were set at 45 lux. They were left to habituate for 15 min before the assessment of mechanical sensitivity threshold using Electronic von Frey (BioSeb/Canada), able to determine the threshold based on the pressure applied on the surface by the experimenter (Martinov et al., 2013). The plastic tip was pressed against the centre of the hind paw until the withdrawal response for a total of three trials per hind paw (right and left). The test was performed 2 days before PTX or VEH administration, and on days 7, 14, 24, 42 and 69 after the first PTX injection (from now on indicated as “post-PTX”).

##### 2.3.1.2 Acetone Drop test

To measure cold hypersensitivity, the test was performed according to (Yoon et al., 1994), i.e., three applications of a cold drop of acetone were made on the centre surface of the hind paws using a 1 mL syringe. Test was performed after von Frey, so on days 7, 14, 24, 42 and 69 post-PTX, on both the left and right hind paws, using the same light conditions (45 lux). Latency to the first paw withdrawal after the drop application and number of responses were recorded within the first 60 s.

##### 2.3.1.3 Hargreaves’ test

As previously described (Ferdousi et al., 2023), to measure heat hypersensitivity, the animals were placed in the apparatus (IITC Life Sci Inc., United States) comprised of a six-chambered acrylic arena (11 × 20 × 15 cm per compartment) positioned on a glass panel to habituate for 15 min with the same light conditions used for the other pain-related tests (45 lux). After the acclimatisation, a radiant light source (active intensity of 30% corresponding to 53°C) was applied from below to the centre surface of the hind paw for up to 20 s or until a hind paw withdrawal, lick or flinch response occurred. The latency to these responses was recorded. The test was performed 1 day before the PTX or VEH administration and on days 8, 15, 25, 43, and 70 post-PTX, on both the left and right hind paws.

#### 2.3.2 Cognition-related behaviour

##### 2.3.2.1 Novel object recognition test

The test was performed on day 28 post-PTX as previously described (Llorente-Berzal et al., 2012) to assess the presence of cognitive impairment. The animals were placed in a circular arena (75 cm diameter, with an aluminium reflective wall 40 cm high) for 5 min for 4 consecutive days with light levels set at 100 lux. On the fifth day, the animals were exposed first to two identical objects (called familiar objects from now on) with which they had to interact for a minimum period of 30 s or a maximum of 4 min. Animals that do not exhibit the minimum interaction time required are excluded from the test. At the end of this phase, they were put back in their home cages. After 4 h, they were re-exposed to one of the familiar objects and a novel object for 3 min. Total time, first 30 and 60 s spent exploring each object were recorded with an overhead camera and analysed with the aid of EthoVision XT software (Noldus Information Technology, Wageningen, Netherlands) and the Discrimination Ratio (DR) was calculated as follows with T being the time spent exploring objects

$$DR=\frac{T_{\left(novel\right)}-T_{\left(familiar\right)}}{T_{\left(total\right)}}$$

##### 2.3.2.2 T-Maze Spontaneous Alternation test

The test was performed on days 35 and 36 post-PTX according to (Deacon and Rawlins, 2006) with some minor changes to better assess the presence of any spatial memory impairment in the model used. The animals were placed into a black-painted T-shaped arena (50 cm in length and 10 cm in width) with 18 lux level. During the first phase (sample phase), animals were placed in the arena with a central partition and left free to choose one of the two arms of the apparatus. After making their choice, a guillotine door slid down, and the animals were kept there for 30 s. In the following phase (choice phase), animals were placed in the arena without the central partition and left free to choose one of the arms for a maximum of 90 s. The test was carried out over 2 days with five exposures per day, and a total number of ten exposures per animal. The percentage of alternation, error in alternation, error of missed choice were analysed for each animal.

#### 2.3.3 Anxiety-related behaviour

##### 2.3.3.1 Elevated Plus Maze test

The test was performed to assess changes in the natural tendency of rodents to avoid open spaces (Kraeuter et al., 2019) on day 49 post-PTX. The arena in which animals were placed was elevated 50 cm above the floor of the testing room, and it consisted of a maze with two arms closed by walls (30 cm high, 50 × 10 cm), two open arms (50 × 10 cm), and a central area that connected the arms (10 × 10 cm). The light levels were at 45 lux in the open arms and 15 lux in the closed arms. Animals were placed in the central platform facing one of the open arms for 5 min, while the time spent in, and the frequency of entries into, the central area of the arena and the open and closed arms were recorded with an overhead camera and analysed with the aid of EthoVision XT software (Noldus Information Technology, Wageningen, Netherlands).

##### 2.3.3.2 Open Field test

Immediately after the Elevated Plus Maze test on day 49 post-PTX, animals were placed in a square arena (43 × 43 × 43 cm) for 5 min to monitor the exploration of different areas of a novel brightly lit apparatus and locomotor activity (Gould et al., 2009). The light levels were at 200 lux. The locomotor activity and time spent in the inner and outer zones were recorded with an overhead camera and analysed with the aid of EthoVision XT software (Noldus Information Technology, Wageningen, Netherlands).

##### 2.3.3.3 Light Dark Box test

The test was performed on day 51 post-PTX (Crawley, 1985). The two-chambered arena was comprised of a light area (30 × 30 cm) illuminated at 150 lux, and a dark area of the same size at 0 lux at the corner of the compartment was defined as the illuminated light chamber at 150 lux. The other compartment was designated as the dark chamber illuminated at 0 lux at the corners and 5 lux at the passage entrance connecting both chambers. Animals were placed in the bright area and left free to explore the apparatus for 20 min. The frequency of entries and time spent in each chamber were recorded with an overhead camera and analysed with the aid of EthoVision XT software (Noldus Information Technology, Wageningen, Netherlands).

#### 2.3.4 Depression-related behaviour

##### 2.3.4.1 Sucrose Preference test

To measure the natural inclination of animals to consume a palatable liquid, deficits in which are considered a measure of depression-related behaviours (Willner et al., 1987), the test was carried out as described in (Boullon et al., 2021) starting on day 54 post-PTX. Briefly, for 4 days the pair-housed animals were trained to drink from a bottle placed on either side of the home cage (right or left), where the light levels were set at 30 lux. On days 1 and 2, the bottle was filled with a solution of 0.5% sucrose, while on days 3 and 4 the bottle was filled with water. The position of the bottle changed from the right to the left every 24 h. On day 5, the animals were provided with a bottle of sucrose 0.5% placed on the right side, and a bottle of water placed on the left. The bottle positions were swapped after 12 h and the test ended after an additional 12 h. At any stage of this test, the bottles were weighed. The preference for sucrose solution was calculated as:

$$\%\;Sucrose\;Preference=\frac{Sucrose\;Intake}{Total\;Intake}mL\times 100$$

Additionally, the sucrose intake was expressed as a percentage of 0.5% sucrose consumed per body weight (Hestehave et al., 2020), calculated as:

$$\text{Percentage of 0.5\% sucrose per body weight}=\frac{\text{Sucrose Intake (mL)}}{\text{Body weight (g)}}\times 100$$

The body weight and the sucrose intake used were the average value between the weights and the consumption of the two animals in the cage, as they were pair-housed.

##### 2.3.4.2 Sucrose splash test

Considering the importance of self-grooming behaviour in rats (Kalueff et al., 2016), this behaviour may be considered a measure of self-care and was analysed using the Sucrose Splash test. The test was carried out on day 62 post-PTX by placing the animals in a Plexiglass box (30 × 30 × 40 cm^3^) at 30 lux for a habituation period of 10 min. After this time, the dorsum of the animals was sprayed with a 10% sucrose solution and the self-grooming behaviour was recorded for 10 min with a camera placed underneath the arena and analysed with the aid of EthoVision XT software (Noldus Information Technology, Wageningen, Netherlands).

##### 2.3.4.3 Forced Swim test

The test was carried out on day 65 post-PTX with the first exposure, and on day 66 post-PTX for the second exposure, according to (Detke et al., 1995), considering immobility as a passive coping strategy that rats adopt after a period of active escape-oriented behaviour. Essentially, the animals were forced to swim for 15 min in Plexiglass cylinders (45 cm height, 20 cm diameter) containing water at 25°C; the light level in the room was set a 30 lux. After 24 h, rats were re-exposed to this environment for 5 min. The exposures were recorded with a camera, and the time spent immobile (absence of movements), climbing (vertical movements), and swimming (horizontal movements) were scored with the aid of EthoVision software XT (Noldus Information Technology, Wageningen, Netherlands).

### 2.4 Tissue collection

Animals were euthanised at day 71 post-PTX by live decapitation. The hypothalamus, prefrontal cortex, amygdala, hippocampus, periaqueductal grey, thalamus, rostral ventral medulla, and dorsal lumbar area of the spinal cord were gross-dissected, snap-frozen on dry ice and stored at −80^o^C prior to measurement of endocannabinoids and related *N*-acylethanolamines (Figure 1). Lateralised regions were harvested separately as left and right.

### 2.5 Measurement of endocannabinoids and related *N*-acylethanolamines by liquid chromatography coupled to tandem mass spectrometry (LC-MS/MS)

Levels of endocannabinoids (AEA, 2-AG) and related *N*-acylethanolamines (PEA, OEA) were measured according to (Boullon et al., 2021; Corcoran et al., 2020; Kerr et al., 2012) in the spinal cord, prefrontal cortex, hypothalamus, amygdala, hippocampus, periaqueductal grey, thalamus, and rostral ventral medulla.

A solution composed of 200 μL of 100% acetonitrile containing deuterated internal standards (2.5 ng d8-AEA, 50 ng d8-2-AG, 2.5 ng d4-PEA, 2.5 ng d4-OEA; Cayman Chemicals, Cambridge Biosciences, UK) was added to each sample, and 75 μL of 100% acetonitrile only. The samples were then homogenised using a sonicator (Sonifier, Branson, Ireland) for ∼5 s and then centrifuged at 14,000 × g for 15 min at 4°C (Hettich^®^ centrifuge Mikro 22R, Hettich, Germany). To calculate the unknown analyte concentration, a 10-point standard curve was made by adding 75 μL of acetonitrile 100% to the higher standard (10), composed of 25 μL of acetonitrile 100% and non-deuterated 25 ng AEA, PEA, OEA and 250 ng 2-AG; the sample was vortexed, and 25 μL of this mixture was removed and placed into the next tube, with serial dilution from the highest to the lower standard (1), discarding the last 25 μL to ensure the same volume in each tube. Once completed, the solution composed of 200 μL of 100% acetonitrile with deuterated internal standard were added to each standard tube. A total amount of 40 μL was removed from sample and standard tubes and transferred to HPLC vials.

A solution comprised of HPLC grade water with 0.1% formic acid, and a solution comprised of 100% acetonitrile with 0.1% formic acid for 3 min were used with a flow rate of 0.2 mL/min using a Waters Atlantis T3 column (3 μm particles, 100 mm length, 2.1 mm diameter; Waters, UK). The elution was carried out using in reverse-phase gradient, starting with 2% acetonitrile for 3 min, followed by an increase to 65% acetonitrile at 3.1 min for 1 min, and then a linear ramp to 100% by 8 min, where it was maintained until 16 min. The initial conditions were then restored at 16.1 min for 12 min to re-equilibrate the column before the next injection.

Detection of analytes was performed using electrospray-positive ionization mode on an Agilent 1100 HPLC system, which was connected to a triple quadrupole 6,460 mass spectrometer (Agilent Technologies, Cork, Ireland). Quantification was achieved ratiometrically with the Agilent Mass-Hunter Quantitative Analysis Software (Agilent Technologies, Cork, Ireland). The analyte concentrations in unknown samples were determined by calculating the peak area response ratio of the analyte to the internal standard.

### 2.6 Measurement of levels of mRNA encoding CB_1_, CB_2_, FAAH, and MGL within the amygdala by real time-quantitative polymerase chain reaction

The total RNA was extracted from pellets following the preparation for LC-MS/MS using a Macherey-Nagel NucleoSpin RNA (Mini Kit for RNA purification, Fisher Scientific, Ireland), as per manufacturer instructions. RNA concentration, integrity, and purity were assessed using a Nanodrop spectrophotometer (ND-1000; Nanodrop, Labtech International, UK). Purity was assessed by the absorbance ratio at 260/280, with values ∼2 considered acceptable, while the 260/230 ratio, with an acceptable range of 2.0–2.2, was used to indirectly assess integrity. The samples were normalised to a concentration of 88 ng/μL. The transcription of the RNA to cDNA was done using a High-Capacity cDNA Reverse Transcription Kit (ThermoFisher Scientific, Ireland). TaqMan gene expression assay was carried out using an Applied Biosystems “StepOne Plus” instrument (Bio-Sciences, Dun Laoghaire, Ireland) using TaqMan Universal PCR Master Mix, no AmpErase UNG (ThermoFisher Scientific, Ireland), and different FAM-labelled probes to quantify the genes of interest (*cnr1* gene for CB_1_, assay ID: Rn00562880_m1; *cnr2* gene for CB_2_, assay ID: Rn03993699_s1; *faah* gene for FAAH, assay ID: Rn00577086_m1; *mgll* gene for MGL, assay ID: Rn00593297_m1) and a VIC-labelled ACTB for beta-actin as housekeeping gene (Assay ID: Rn00667869_m1).

### 2.7 Statistical analysis

IBM SPSS Statistics 27.0 statistical software (Chicago, United States) was used to perform the statistical analysis and GraphPad Prism 10.2.3 (GraphPad Software, Boston, Massachusetts United States) for graph design. Normality and homogeneity were assessed by Shapiro-Wilk test and Levene’s test, respectively. When parametric assumptions were met, datasets were analysed either with a standard two-way Analysis of Variance (ANOVA) (factors: Sex, Treatment) followed by Tukey HSD (Honest Significant Difference) *post hoc* test for multiple comparisons or a two-way repeated measures ANOVA followed by Tukey’s HSD. Three-way ANOVA (Factor: Sex, Treatment, Side) was used to analyse levels of endocannabinoids and *N*-acylethanolamines in lateralised regions (spinal cord, hippocampus, and amygdala). When parametric assumptions were not met, datasets were analysed by Friedman’s two-way ANOVA by ranks followed by Mann–Whitney U *post hoc* with Bonferroni-Holm correction to assess differences between groups at each specific time point. If significant main effects of PTX or sex were observed, or their interaction, then *a priori post-hoc* pairwise group comparisons were carried out. Parametric data are expressed as group means ± standard error of the mean (±SEM) and non-parametric data as medians with interquartile range, and for all the significance level was set at *p < 0.05*.

## 3 Results

Tables 1, 2 summarise the results of the behavioural assays performed relative to the days post-PTX and the results of the *post-mortem* analysis, respectively. Data on body weight throughout the study are presented in Supplementary Figure S1.

**TABLE 1:** Summary table of the results of behavioural assays performed relative to the days post-PTX.

Days post-PTX																		
Behaviour	Assay	7	8	14	15	24	25	28	35	42	43	49	51	54	62	65	69	70
Pain-related	Von Frey	+		+		+				+							+	
	Acetone Drop	+		+		+				+							+	
	Hargreaves'		−		−		−				−							−
Cognition-related	Novel Object Recognition							−										
	T-Maze Spontaneous Alternation								−									
Anxiety-related	Elevated Plus Maze											−						
	Open Field											−						
	Light Dark Box												−					
Depression-related	Sucrose Preference													−				
	Sucrose Splash														−			
	Forced Swim															−		

(+) indicates a significant effect of PTX; (−) indicates absence of effect; empty cell indicates that the test was not performed at that time point.

**TABLE 2:** Summary table of the results of *post-mortem* analysis performed.

Targets									
Analysis	Region	AEA	PEA	OEA	2-AG	CB₁	CB₂	MAGL	FAAH
LC-MS/MS	Hypothalamus	−	−	−	−				
	Prefrontal Cortex	−	−	−	−				
	Thalamus	−	−	−	−				
	Periaqueductal grey	−	−	−	−				
	Rostral ventral medulla	−	−	−	−				
	Hippocampus	−	−	−	−				
	Amygdala	−	+	+	+				
	Spinal Cord	−	−	−	−				
RT-qPCR	Amygdala					−	−	−	−

(+) indicates a significant effect of PTX; (−) indicates absence of effect; empty cell indicates that the test was not performed.

### 3.1 PTX induced long-term mechanical hypersensitivity in SD rats of both sexes

**FIGURE 2:**
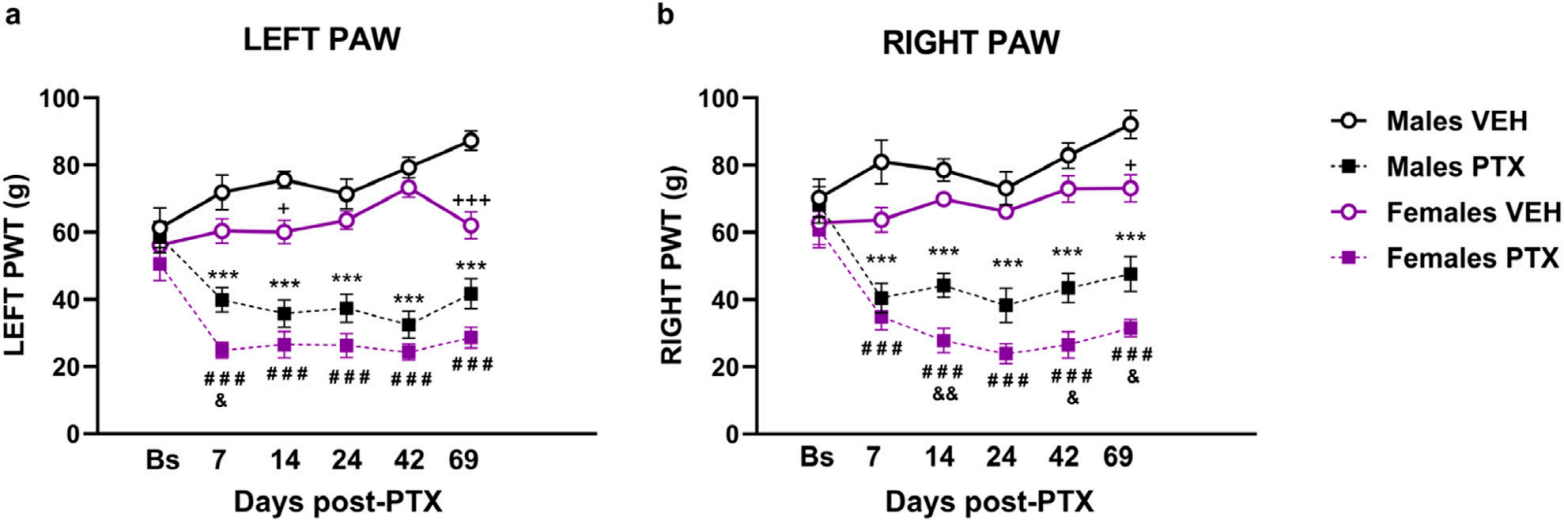
Von Frey test. Investigation of mechanical hypersensitivity in male and female rats following Paclitaxel (PTX) or vehicle (VEH) intraperitoneal injections from Baseline (Bs) to day 69. Paw withdrawal threshold (PWT): left paw **(A)** and right paw **(B)**. Two-way repeated measures ANOVA followed by Tukey HSD (Honest Significant Difference) *post hoc*. Data expressed as mean ± S.E.M (n = 10 per group) *p < 0.05, **p < 0.01, ***p < 0.001 Males PTX vs. Males VEH, *#p < 0.05, ##p < 0.01, ###p < 0.001* Females PTX vs. Females VEH, and *p < 0.05*, and *p < 0.01* Females PTX vs. Males PTX, + *p < 0.05, +++ p < 0.001* Females VEH vs. Males VEH.

There were no significant between-group differences in hind paw withdrawal thresholds (PWT) to mechanical stimulation (electronic von Frey) at baseline. Significant differences between groups in mechanical hypersensitivity, measured as paw withdrawal thresholds (PWT), were observed at every post-PTX time point analysed for both hind paws (Figures 2A, B). Two-way ANOVA with repeated measures with sex and treatment as factors for both paws revealed a significant effect of time (Left: F_(3.58,128.74)_ = 3.791; *p* = 0.008. Right: F_(3.71,133.52)_ = 8.591; *p* < 0.001), a significant effect of the interaction Time × Treatment (Left paw: F _(3.58,128.74)_ = 3.791; *p <* 0.001 Right paw: F_(3.71,133.52)_ = 18.670; *p <* 0.001), and a significant effect of Sex (Left: F_(1,36)_ = 18.886; *p* < 0.001. Right: F_(1,36)_ = 18.047; *p* < 0.001) and Treatment (Left: F_(1,36)_ = 160.148; *p* < 0.001. Right: F_(1,36)_ = 134.784; *p* < 0.001).

*Post hoc* analysis revealed that PTX induced a decrease in PWT in the left (Figure 2A) and right (Figure 2B) hind paws in both sexes at each time point analysed, compared with VEH-treated controls. Females treated with PTX exhibited significantly lower left hind paw PWT on day 7, and significantly lower right hind paw PWT on days 14, 42 and 69, compared with male counterparts. However, females treated with VEH also exhibited significantly lower left hind paw PWT on days 14 and 69, and significantly lower right hind paw PWT on day 69, compared with male counterparts.

### 3.2 PTX-induced cold hypersensitivity long-term in SD rats of both sexes

**FIGURE 3:**
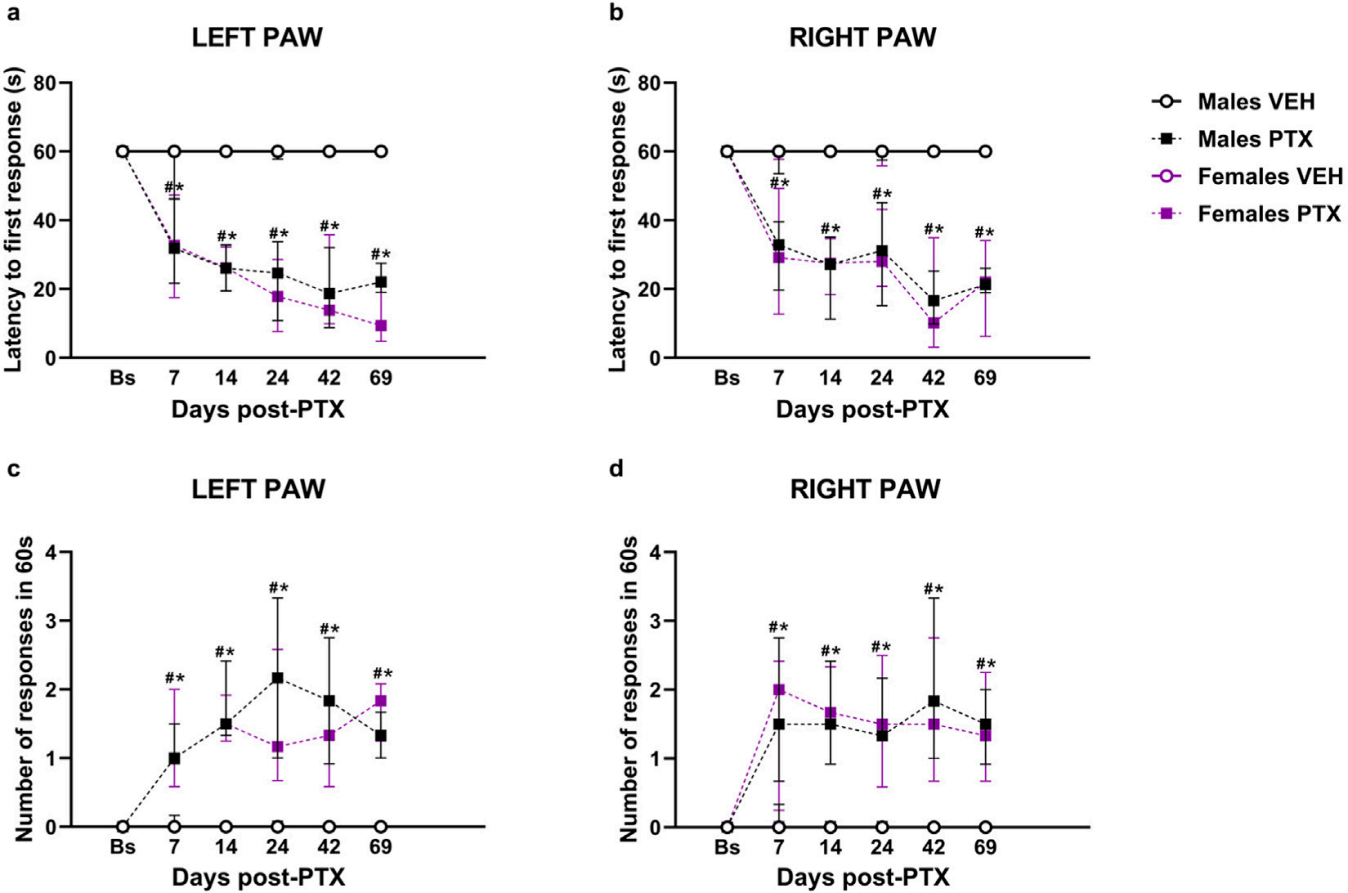
Acetone Drop test. Investigation of cold hypersensitivity in male and female rats following PTX or VEH intraperitoneal injections. Left **(A)** and right **(B)** paw latency to the first response after acetone stimulation and left **(C)** and right paw **(D)** number of responses after acetone stimulation. Data expressed as Median with IQR (n = 10 per group). Friedman’s two-way ANOVA by ranks followed by Mann–Whitney U *post hoc* with Bonferroni-Holm correction, *p < 0.05 Males PTX vs. Males VEH, #*p < 0.05* Females PTX vs. Females VEH, and *p < 0.05* Females PTX vs. Males PTX.

Friedman’s test revealed significant differences between groups in cold hypersensitivity, measured as latency to the first response in seconds, in both hind paws (Left Paw Latency: χ^2^ (5) = 52.693, *p < 0.001,* Figure 3A; Right Paw Latency: χ^2^ (5) = 35.315, *p < 0.001,* Figure 3B). *Post hoc* analysis revealed that PTX induced a decrease in the latency to the first response in the left (Figure 3A) and right (Figure 3B) hind paw in both sexes at each time point analysed, compared to VEH-treated controls and compared with baseline. No significant differences were observed between the other groups.

Furthermore, significant differences between groups were found when measuring cold hypersensitivity as the number of responses within 60 s, at every time point analysed in left (χ^2^ (5) = 46.803, *p < 0.001,* Figure 3C) and right (χ^2^ (5) = 37.675, *p < 0.001,* Figure 3D) hind paws. *Post hoc* analysis revealed that PTX induced an increase in the number of responses within 60 s on the left (Figure 3C) and right (Figure 3D) hind paws in both sexes at each time point analysed, compared to VEH-treated controls and compared with baseline. No significant differences were observed in the other groups.

### 3.3 PTX did not induce heat hypersensitivity in SD rats of both sexes

**FIGURE 4:**
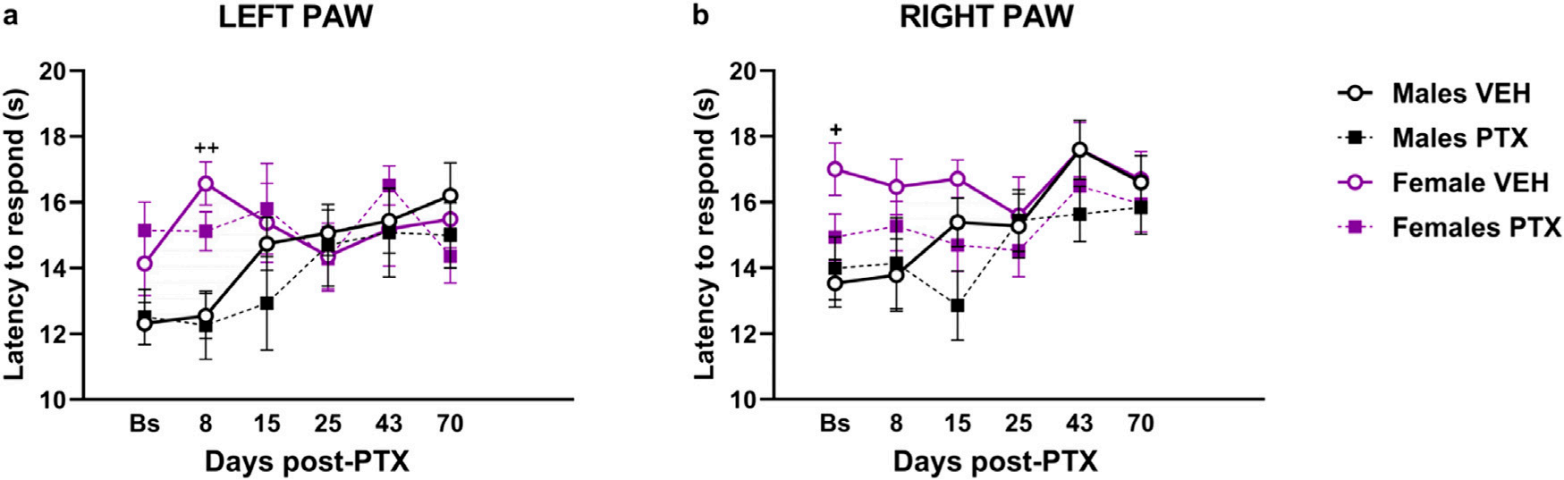
Hargreaves’ test. Investigation of heat hypersensitivity in male and female rats following Paclitaxel (PTX) or vehicle (VEH) intraperitoneal injections from Baseline (Bs) to day 70. Left **(A)** and right **(B)** latency to the first response after heat stimulation. Two-way repeated measure ANOVA followed by Tukey HSD (Honest Significant Difference) *post hoc*. Data expressed as mean ± S.E.M (n = 10 per group) *+ p < 0.05, ++ p < 0.01* Females VEH vs. Males VEH.

Significant differences between groups were found in heat sensitivity, measured as latency to the first response within 20 s at different time points analysed in both paws (Figures 4A, B). Two-way ANOVA with repeated measures with sex and treatment as factors revealed a significant effect of time for both paws (Left: F_(3.58,128.74)_ = 3.791; *p* = 0.008. Right: F_(3.71,133.52)_ = 8.591; *p* < 0.001) and a significant effect of the interaction of Time × Treatment for both paws (Left paw: F_(5,180)_ = 3.509; *p =* 0.005. Right paw: F_(5,180)_ = 5.152; *p <* 0.001). *Post hoc* analysis revealed that PTX did not induce any changes in the latency to the first response in the left (Figure 4A) and right (Figure 4B) in both sexes at each time point analysed, compared with VEH-treated controls or compared with baseline. However, females treated with VEH showed a significant increase in paw latency compared to their male counterparts, on the left paw latency on day 8, and on the right paw latency at the Baseline.

### 3.4 PTX did not alter cognition-related behaviour in SD rats of both sexes

**FIGURE 5:**
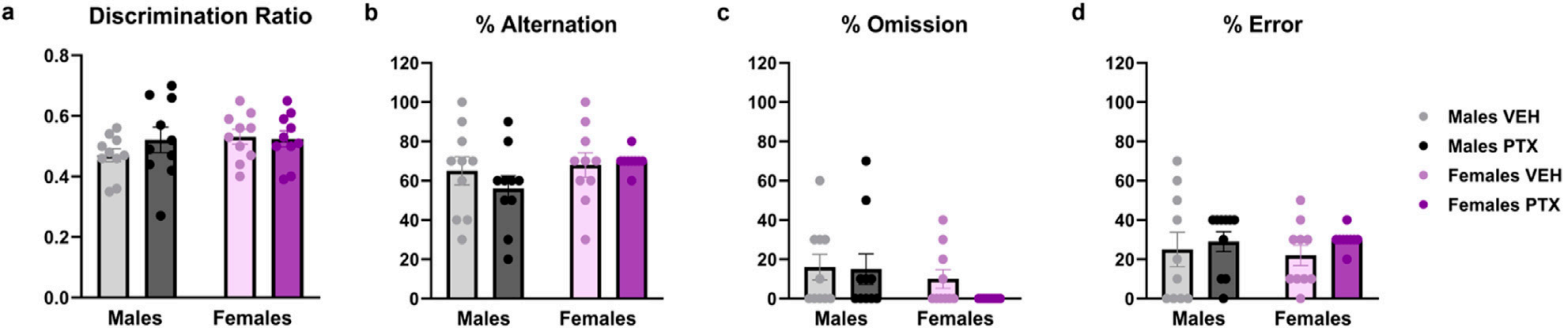
Novel Object Recognition and T-Maze Spontaneous Alternation tests. Investigation of cognition-related behaviour in male and female rats on days 28 (Novel Object Recognition test) and 35 (Alternated T-Maze test) following PTX or VEH intraperitoneal injections. The ratio **(A)** was calculated as Discrimination Ratio = (Time spent exploring the novel object - Time spent exploring the familiar object)/Total time spent exploring the objects. The percentage of alternation **(B)**, omission **(C)**, and error **(D)** in the T-Maze Spontaneous Alternation test. Data expressed as mean ± SEM (n = 10 per group).

No significant differences between groups were found in Novel Object Recognition test (Figure 5A; Supplementary Figures S2A–S2C) or T-Maze Spontaneous Alternation test (Figures 5B–D).

### 3.5 PTX did not alter anxiety-related behaviour in SD rats of both sexes

**FIGURE 6:**
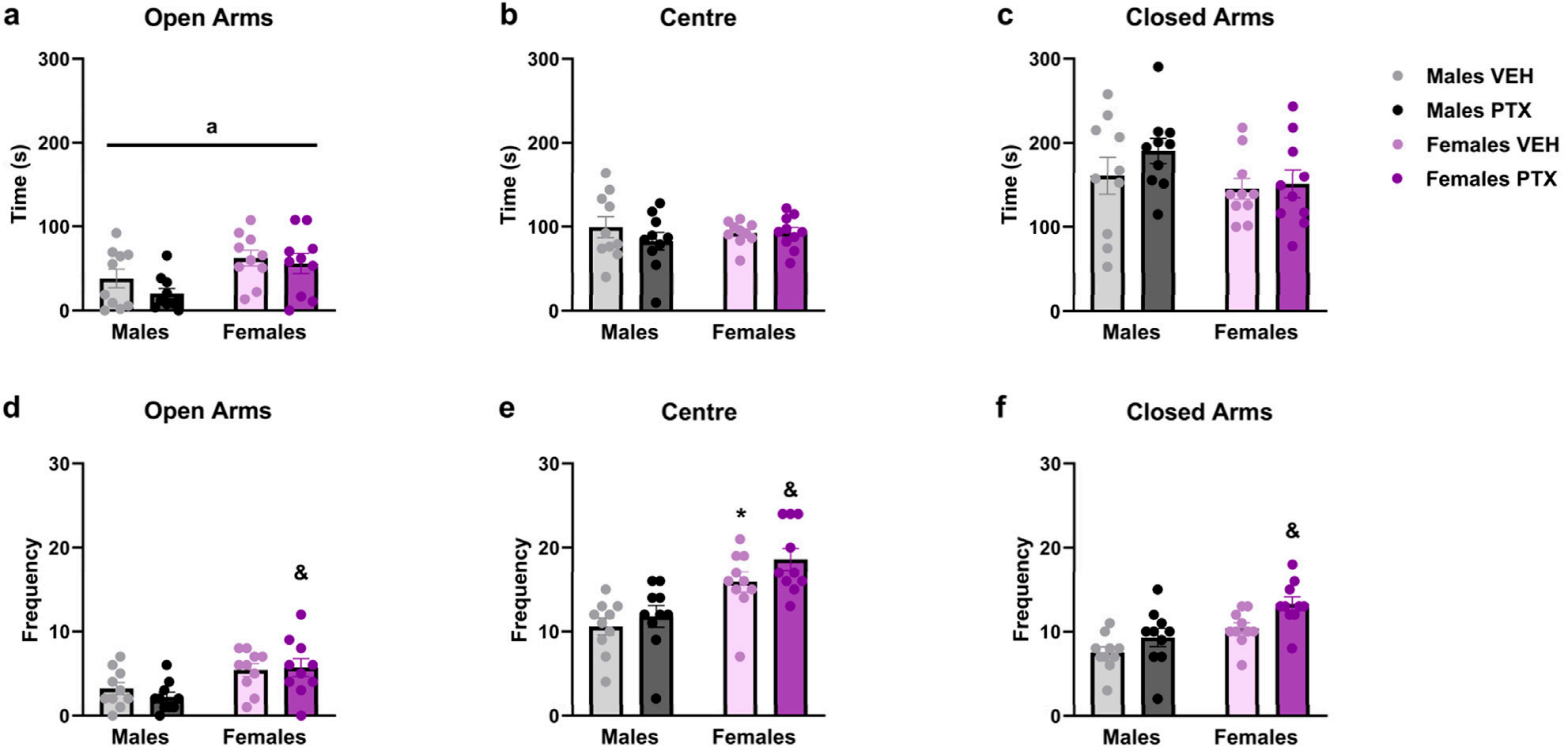
Elevated Plus Maze test. Investigation of anxiety-related behaviour in male and female rats on day 49 following PTX or VEH intraperitoneal injections. Time spent in open arms **(A)**, central area of the apparatus **(B)**, and closed arms **(C)**. Frequency of entries in open arms **(D)**, central area of the apparatus **(E)**, and closed arms **(F)**. Two-way ANOVA followed by Tukey HSD (Honest Significant Difference) *post hoc*. Data expressed as mean ± S.E.M (n = 10 per group). *p < 0.05 Males PTX vs. Males VEH, and *p < 0.05* Females PTX vs. Males PTX, a = significant effect of sex in ANOVA.

PTX had no significant effects on behaviour in the Elevated Plus Maze (Figure 6A–F). However, a significant ANOVA effect for the factor sex emerged in the time spent in the open arms (Figure 6A), with an increase for females treated either with PTX or VEH (Time spent in the open arms: F_(1,36)_ = 9.204; *p* = 0.004). Regarding the frequency of entries into the different areas of the apparatus: there was a significant increase in: entries in open arms displayed by females treated with PTX when compared to their male counterparts (Figure 6D) (Frequency of entries into open arms: F_(1,36)_ = 12.842; *p* = 0.001); entries in the central area of the apparatus where females treated either with PTX or VEH showed a significant increase when compared to their male counterparts (Figure 6E) (Frequency of entries into the central area: F_(1,36)_ = 24.989; *p* < 0.0010); entries in the closed arms where females treated with PTX showed a significant increase when compared to their male counterparts (Figure 6F) (Frequency of entries in the closed arms: F_(1,36)_ = 7.855; *p* = 0.008).

**FIGURE 7:**
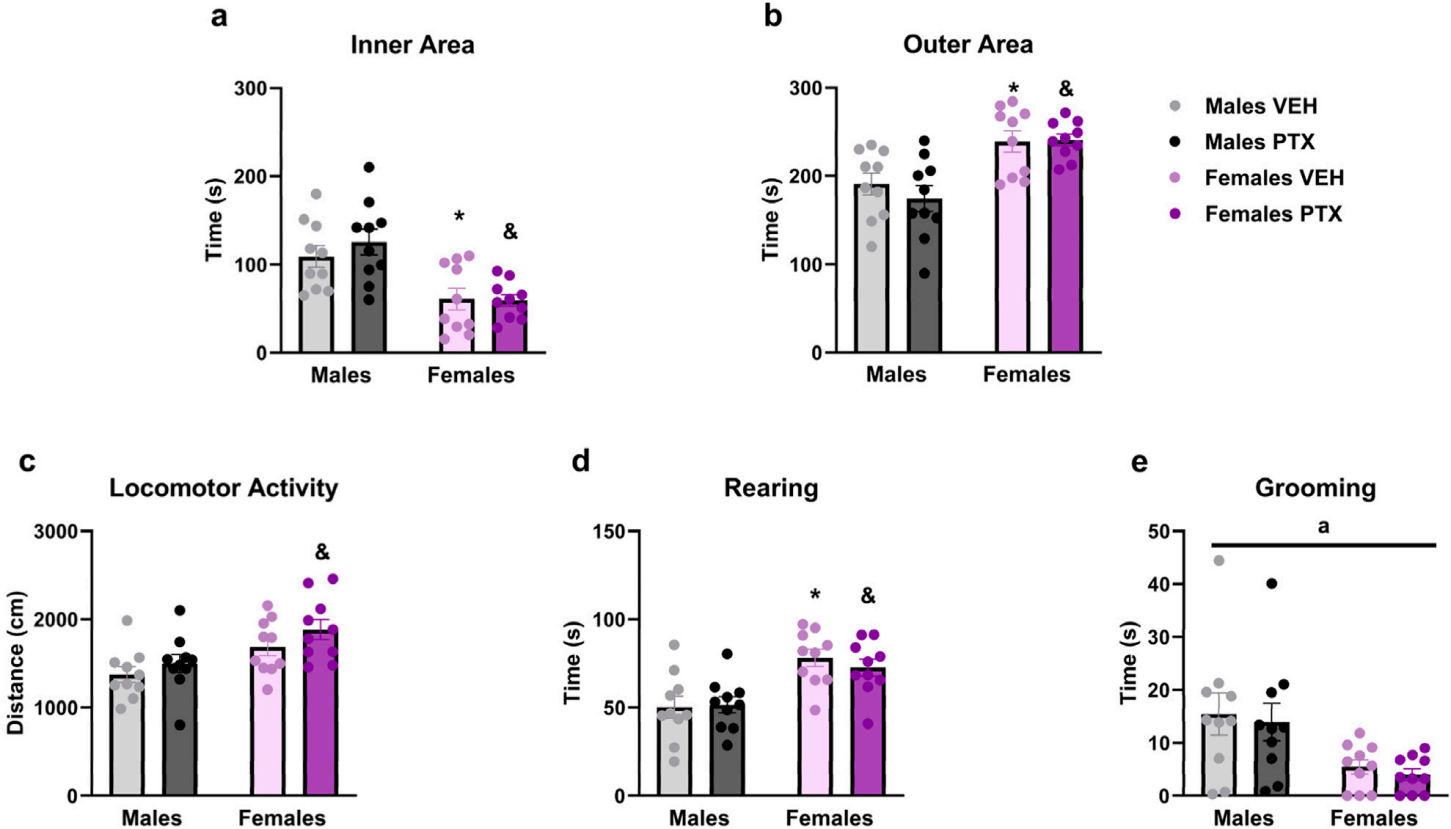
Open field test. Investigation of anxiety-related behaviour in male and female rats on day 49 following PTX or VEH intraperitoneal injections. Time spent in the inner area of the apparatus **(A)**, outer area **(B)**, locomotor activity **(C)**, rearing **(D)**, and grooming **(E)**. Two-way ANOVA followed by Tukey HSD (Honest Significant Difference) *post hoc*. Data expressed as mean ± S.E.M (n = 10 per group). *p < 0.05 Males PTX vs. Males VEH, and *p < 0.05* Females PTX vs. Males PTX, a = significant effect of sex in ANOVA.

PTX had no significant effects on behaviour in the Open field test. However, there were significant main effects of sex on time spent in the inner area of the apparatus (Figure 7A) (F_(1,36)_ = 23.717; *p* < 0.001), time spent in the outer area of the apparatus (Figure 7B) (F_(1,36)_ = 23.707; *p* < 0.001), distance moved (Figure 7C) (F_(1,36)_ = 11.937; *p* = 0.001), time spent rearing (Figure 7D) (F_(1,36)_ = 22.836; *p* < 0.001) and time spent grooming (Figure 7E) (F_(1,36)_ = 12.566; *p* = 0.001). Further *post hoc* analysis confirmed that females treated either with VEH or PTX displayed increased time in the inner area, outer area, rearing and grooming, compared to their male counterparts. Females treated with PTX exhibited an increase in distance moved compared to their male counterparts.

**FIGURE 8:**
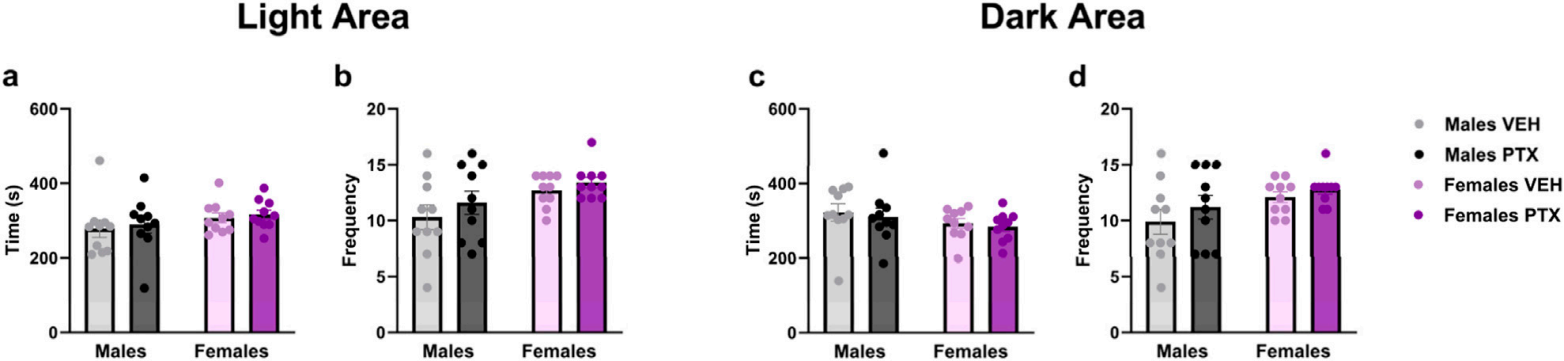
Light Dark Box. Investigation of anxiety-related behaviour in male and female rats on day 51 following PTX or VEH intraperitoneal injections. Time **(A)** and frequency **(B)** in the light area, and time **(C)** and frequency **(D)** in the dark area. Data expressed as mean ± S.E.M (n = 10 per group).

No significant differences between groups were observed in Light Dark Box test (Figures 8A–D).

### 3.6 PTX had no effects on depression-related behaviour in SD rats of both sexes

**FIGURE 9:**
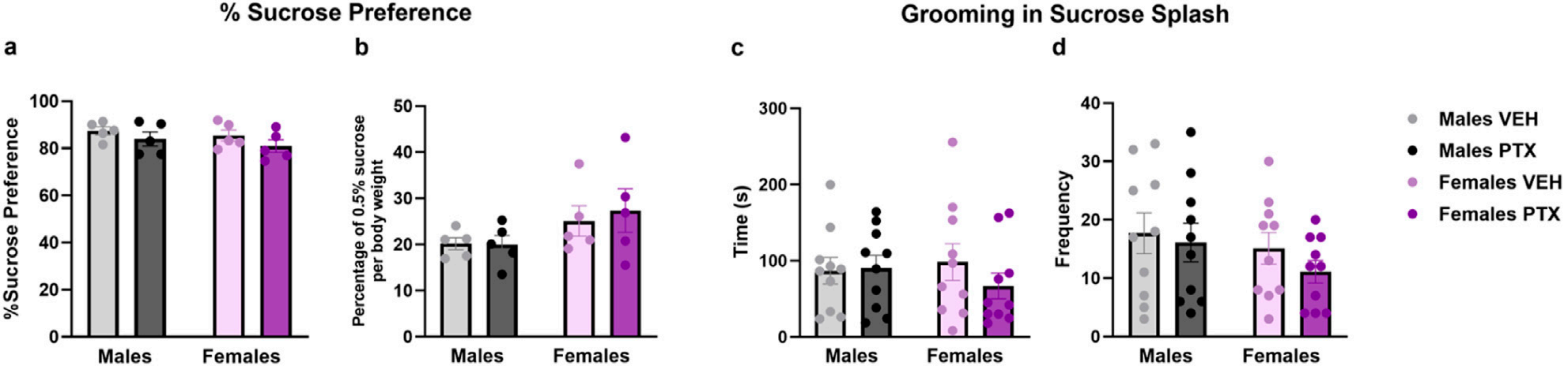
Sucrose preference and Sucrose Splash. Investigation of depression-related behaviour in male and female rats respectively on day 54 and 62 following PTX or VEH intraperitoneal injections. Percentage of Sucrose preference calculated as (Sucrose Intake/Total Intake) × 100 **(A)**, and Percentage of 0.5% sucrose per body weight calculated as (Sucrose Intake)/(Body weight) × 100 **(B)**. Data expressed as mean ± S.E.M (n = 5 per group). Time spent grooming **(C)**, and frequency **(D)** in sucrose splash. Data expressed as mean ± S.E.M (n = 10 per group).

**FIGURE 10:**
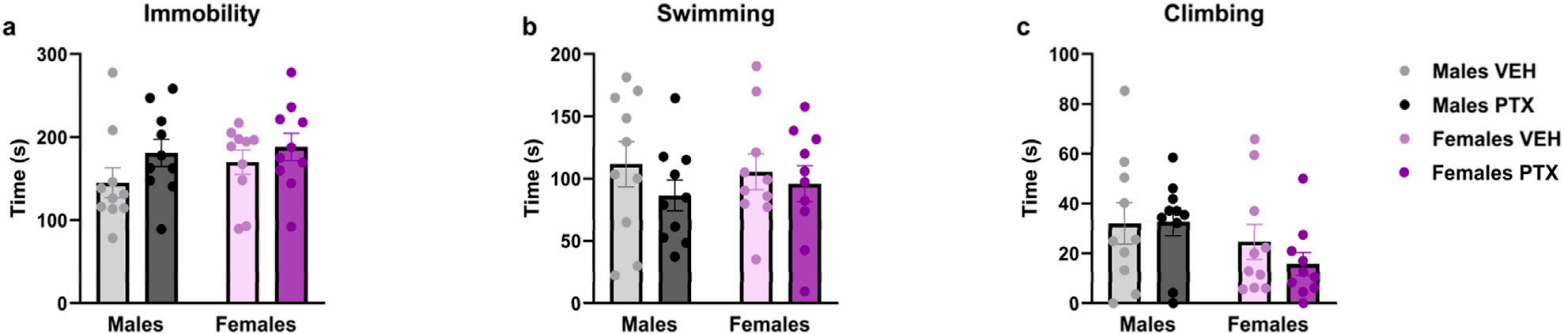
Forced Swim test. Investigation of learned helplessness or behavioural despair in male and female rats on day 65 following PTX or VEH intraperitoneal injections. Time spent immobile **(A)**, swimming **(B)**, and climbing **(C)**. Data expressed as mean ± S.E.M (n = 10 per group).

No significant differences between groups were found in the Sucrose Preference (Figures 9A, B), Sucrose Splash (Figures 9C, D), or Forced Swim (Figures 10A–C; Supplementary Figures S3A–S3C) tests.

### 3.7 PTX induced an increase in PEA, OEA, and 2-AG in the amygdala but not in the other regions in SD rats of both sexes

**FIGURE 11:**
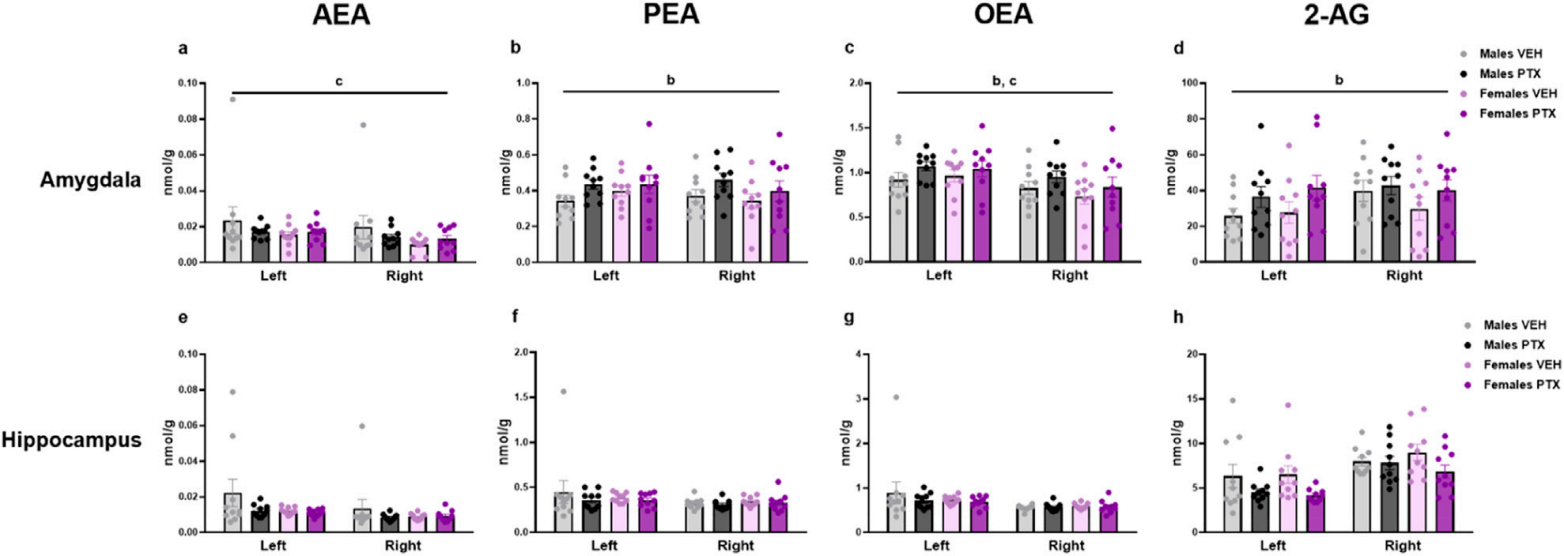
Investigation of levels of endocannabinoids and related *N*-acylethanolamines (nmol/g of tissue) in male and female rats following intraperitoneal injection of VEH or PTX in the amygdala **(A–D)**, and hippocampus **(E–H)**. Three-way ANOVA (factors: sex, treatment, side). Data expressed as mean ± S.E.M (n = 10 per group). b = significant effect of treatment; c = significant effect of side in ANOVA.

**FIGURE 12:**
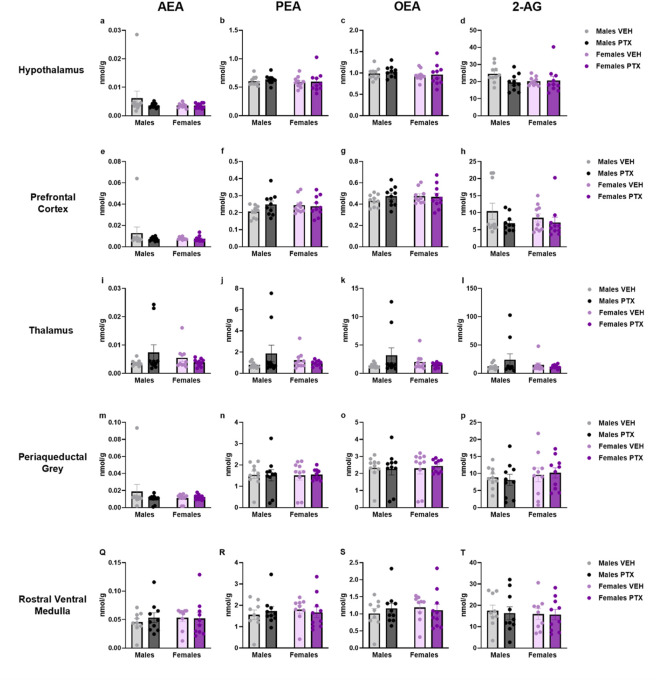
Investigation of endocannabinoid ligands and related *N*-acylethanolamine levels (nmol/g of tissue) in male and female rats following VEH or PTX intraperitoneal injections in the hypothalamus **(A–D)**, prefrontal cortex **(E–H)**, thalamus **(I–L)**, periaqueductal grey **(M–P)**, rostral ventral medulla **(Q–T)**. Data expressed as mean ± S.E.M (n = 10 per group).

**FIGURE 13:**
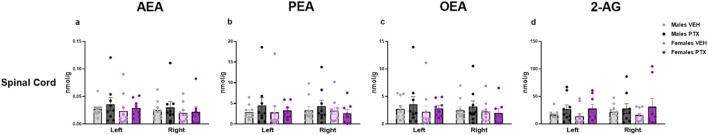
Investigation of endocannabinoid ligands **(A–D)** and related N-acylethanolamine levels **(B,C)** (nmol/g of tissue) in male and female rats following VEH or PTX intraperitoneal injections in the spinal cord. Data expressed as mean ± S.E.M (n = 10 per group).

The measurement of endocannabinoids and related *N*-acylethanolamines in various brain regions and spinal cord revealed a main effect of PTX on 2-AG (F_(1,72)_ = 6.108; *p* = 0.016), PEA (F_(1,72)_ = 12.842; *p* = 0.001), and OEA (F_(1,72)_ = 4.158; *p* = 0.005) in the amygdala, in which also an effect of side was found for AEA (F_(1,72)_ = 10.271; *p* = 0.002) and OEA (F_(1,72)_ = 8.257; *p* = 0.005) (Figures 11A–D). Further *post hoc* analyses did not reveal any significant pairwise between-group differences. No significant differences between groups were found in the other brain regions analysed (hypothalamus (Figures 12A–D), prefrontal cortex (Figures 12E–H), hippocampus (Figures 11E–H), thalamus (Figures 12I–L), periaqueductal grey (Figures 12M–P), rostral ventral medulla (Figures 12Q–T) or spinal cord (Figures 13A–D)).

**FIGURE 14:**
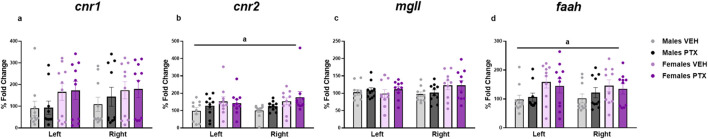
Levels of mRNA for *cnr1* **(A)**, *cnr2* **(B)**, *mgll* **(C)**, *and faah* **(D)**, respectively coding for CB_1_, CB_2_, MGL, and FAAH, in male and female in the amygdala of rats following intraperitoneal injection of VEH or PTX. Three-way ANOVA (factors: sex, treatment, side). Data expressed as mean ± S.E.M (n = 10 per group). a = significant effect of sex in ANOVA.

RT-qPCR on the amygdala tissue revealed a main effect of sex with females showing an increased expression of *cnr2* (F_(1,72)_ = 9.491; *p* = 0.003) and *faah* (F_(1,72)_ = 7.283; *p* = 0.009). However, the *post hoc* analysis did not reveal any significant pairwise between-group differences (Figures 14A–D).

## 4 Discussion

There is a paucity of published studies investigating sex differences in animal models of CIPN (Currie et al., 2019). Such studies are required to advance the understanding and personalised treatment of this condition. Here, we show that PTX administration induced a robust and sustained neuropathic pain-related phenotype in both male and female Sprague-Dawley rats. PTX-treated animals of both sexes exhibited mechanical and cold hypersensitivity over 70 days post-PTX. Paw withdrawal thresholds to mechanical stimulation were lower in PTX-treated female rats than in male counterparts, however, VEH-treated females also exhibited lower withdrawal thresholds than VEH-treated males at discrete time points. The tendency for female rats to exhibit lower paw withdrawal thresholds than males to mechanical stimulation has been already published (Hendrich et al., 2012), consistent with the findings in this study. In addition, it has been reported that hypersensitivity induced by PTX can be more robust in female versus male mice (Ward et al., 2011). While few studies to date have analysed sex differences in the PTX model or in any other CIPN model, the data obtained from male animals in this study is consistent with the majority of studies to date in the PTX-induced neuropathic pain model (Toma et al., 2017) and in other CIPN models such as oxaliplatin (Noya-Riobó et al., 2023). On the other hand, they highlight the need for inclusion of both sexes in future studies to increase the understanding of sex differences in neuropathic pain.

PTX had no effect on sensitivity to noxious heat assessed with the Hargreaves’ test, as previously reported in other publications in which the same strain has been used (Griffiths et al., 2018; Huynh et al., 2019). This is also in line with findings for the same model in mouse, where the same cumulative dose of this study was used (Smith et al., 2004), and it may be related to the absence of neurodegeneration observed with this regimen (Flatters and Bennett, 2006).

Between days 28 and 35 post-PTX, cognition-related behaviour was tested with the Novel Object Recognition and T-Maze Spontaneous Alternation tests, and the animals did not exhibit any PTX-induced alterations in cognitive performance in these tests. However, the VEH-treated groups did not show the expected degree of preference for the novel object under the conditions employed in this study, making it very difficult to draw any firm conclusions relating to the effects of PTX on object recognition memory in this study. The protocol used has previously been used successfully in Wistar rats (Llorente-Berzal et al., 2012), however Sprague-Dawley rats were used in the present study and this strain has previously exhibited lower novel object recognition performance compared to other strains (Gökçek-Saraç et al., 2015). Regardless, the absence of cognitive impairment has been seen in another CIPN model: animals treated with oxaliplatin did not display any treatment effect in the Novel Object Recognition test (Fardell et al., 2015). Moreover, in Sprague-Dawley, PTX has been shown to impair reversal learning without affecting prior learning, new learning, and episodic memory (Panoz-Brown et al., 2017), suggesting that the mechanism underlying this impairment may be both distinctive and highly specific.

The battery of anxiety-related tests was performed between days 49 and 51 post-PTX, and PTX treatment did not alter anxiety-related behaviour in any of the tests performed. However, a sex difference was observed in both the Elevated Plus Maze and the Open Field tests, with females being more active and spending more time in the open arms and central areas compared to males, which is corroborating previous work (Börchers et al., 2022). No between-group differences were detected in the Light Dark Box test; however, animals did not exhibit the expected preference for the dark area of the apparatus versus the light area. It is possible that light levels, which were set at 150 lux in the light chamber, may not have been sufficiently aversive in the Sprague-Dawley strain (Campos-Cardoso et al., 2023). Nevertheless, these affective behaviours seem more evident in mice rather than in rats, where animals showed anxiety-like behaviours in the Elevated Plus Maze, Open Field and Light Dark Box tests (Toma et al., 2017; Liu et al., 2022).

The battery of depression-related tests was conducted between days 54 and 65 post-PTX, and PTX had no effects in any of the tests carried out. To our knowledge, data on these behaviours in the PTX-induced neuropathic pain model in rats have not been documented in the existing literature to date. Effects induced by PTX have been reported in mice for anhedonia and learned helplessness or behavioural despair, respectively after one and 2 weeks from the induction of the model, with animals exhibiting higher time spent immobile in the Forced Swim test and a marked preference for the sucrose solution in the Sucrose preference (Toma et al., 2017). However, an absence of PTX-induced changes within the same tests, strain and species has also been reported (Huehnchen et al., 2017).

Taken together, these results indicate that it is difficult to draw any firm conclusions on anxiety- and depression-related behaviour considering the paucity of studies published so far. More research is needed to fully determine conclusively whether impairments are present in different species, sexes and at different time points. In fact, it is possible that alterations in these behaviours are not present at the time points used in this study but may be found at other time points relative to PTX injection.

On the other hand, the pain behaviour post-PTX appeared to be robust and sustained over time. The underlying mechanisms may involve the endocannabinoid system due to its key role in modulating pain through the descending pain pathway, which includes the periaqueductal grey, rostral ventromedial medulla, and spinal cord. In those areas, CB_1_ receptors are widely expressed and their activation modulates the nociceptive transmission (Hendrich et al., 2012). In the context of CIPN, the potential of the endocannabinoid system as a therapeutic target is well-documented (Deng et al., 2012; Curry et al., 2018), but the role of the endocannabinoid system, specifically in PTX-induced neuropathic pain and associated sexual dimorphism, requires further study.

Our findings indicate that at the time points and conditions employed herein, there were very few effects of PTX on levels of endocannabinoids and related *N-*acylethanolamines in the regions investigated. The only significant change occurred in the amygdala, where we observed a PTX-induced increase in levels of PEA, OEA, and 2-AG. As seen in the spinal nerve ligation model of neuropathic pain, the increase in the endocannabinoid and *N-*acylethanolamine levels may be a response to the pain condition (Mitrirattanakul et al., 2006). Alterations in the endocannabinoid system have been already reported in CIPN models induced by cisplatin, in which 2-AG and AEA levels increased in the lumbar spinal cord (Guindon et al., 2013; Khasabova et al., 2014), but not in the brain (Khasabova et al., 2014).

Regardless, while changes in the endocannabinoid system within the amygdala have been observed in this model, the gross dissection method used in this study limits our ability to draw conclusions on this matter. This procedure does not allow us to determine whether the changes were restricted to one or more amygdalar subdivisions, and further investigation is warranted.

Within the whole amygdala, the analysis of the expression of mRNA for the endocannabinoid system components (receptors and enzymes that catabolise endocannabinoids and *N*-acylethanolamines) was performed, but PTX did not elicit any changes. Nevertheless, it must be noted that there was an effect of sex on the expression of the genes encoding CB_2_ and FAAH; in fact, females exhibited a higher expression of mRNA for these genes when compared to males. To date, investigation of sex differences in the endocannabinoid system has been focused mostly on CB_1_ receptors, with greater expression of mRNA in males compared to females in several regions, including the amygdala (Castelli et al., 2014). There are fewer studies of CB_2_ expression in the brain, however, sexual dimorphism in CB_2_ may mediate a sex difference in glial cell genesis during amygdalar development (Krebs-Kraft et al., 2010). Data provided in this study not only help to fill the existing gap in knowledge, but also provide further evidence of sexual dimorphism in the endocannabinoid system.

In conclusion, this study represents a comprehensive behavioural characterisation of the PTX model of CIPN in adult Sprague-Dawley rats of both sexes. The data indicate long-lasting PTX-induced mechanical and cold hypersensitivity in female and male rats and extend the characterisation of the effects of PTX alone, in the absence of a tumour. Furthermore, the results advance our understanding of sex as a variable in behavioural outcomes and the role of the endocannabinoid system in this model.

## Data Availability

The raw data supporting the conclusions of this article will be made available by the authors, without undue reservation.
