# Supplementary material for: Characterisation of the effects of the chemotherapeutic agent paclitaxel on neuropathic pain-related behaviour, anxiodepressive behaviour, cognition, and the endocannabinoid system in male and female rats
Source: Front Pharmacol. 2025 Jan 3;15:1505980. doi: 10.3389/fphar.2024.1505980 (PMC11739114; doi:10.3389/fphar.2024.1505980)
Supplement: Supplementary file 1 [file DataSheet1.docx]

**Supplementary material**


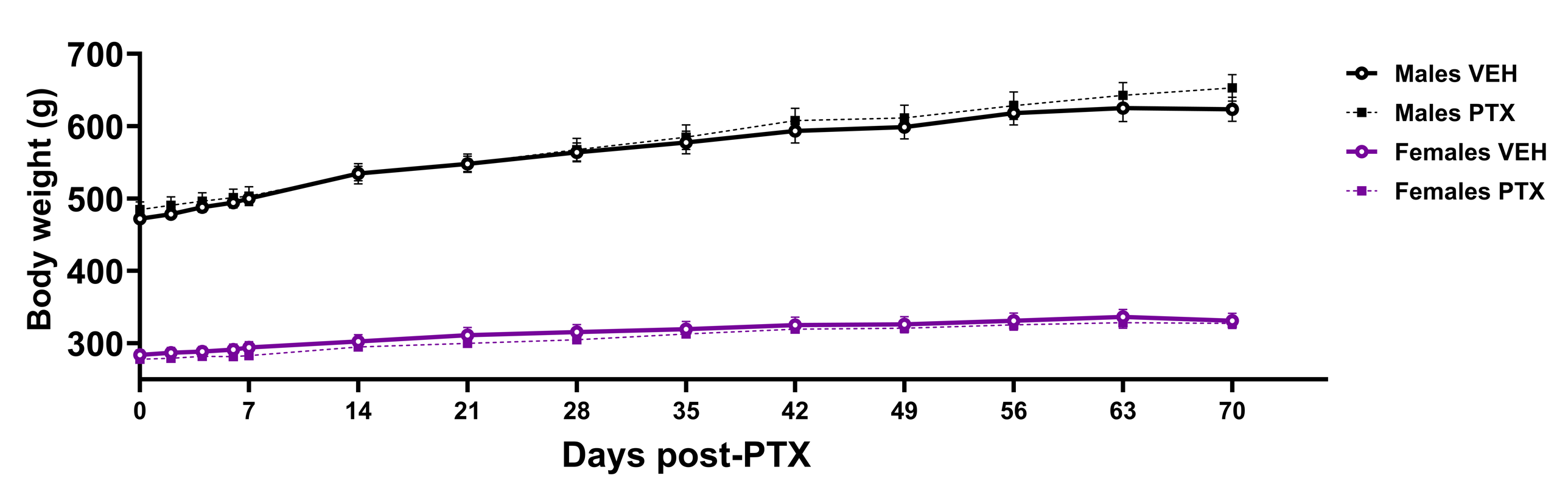
 **Figure S1.** Body weight (g) from the first injection of PTX. Data expressed as mean ± SEM (n=10 per group).



**Figure S2.** Novel Object Recognition test. Exploration of familiar (a) and novel (b) objects during the entire test; exploration of familiar (c) and novel (d) objects during the first minute, and exploration of familiar (e) and novel (f) objects during the first 30 seconds. Data expressed as mean ± SEM (n=10 per group).

**
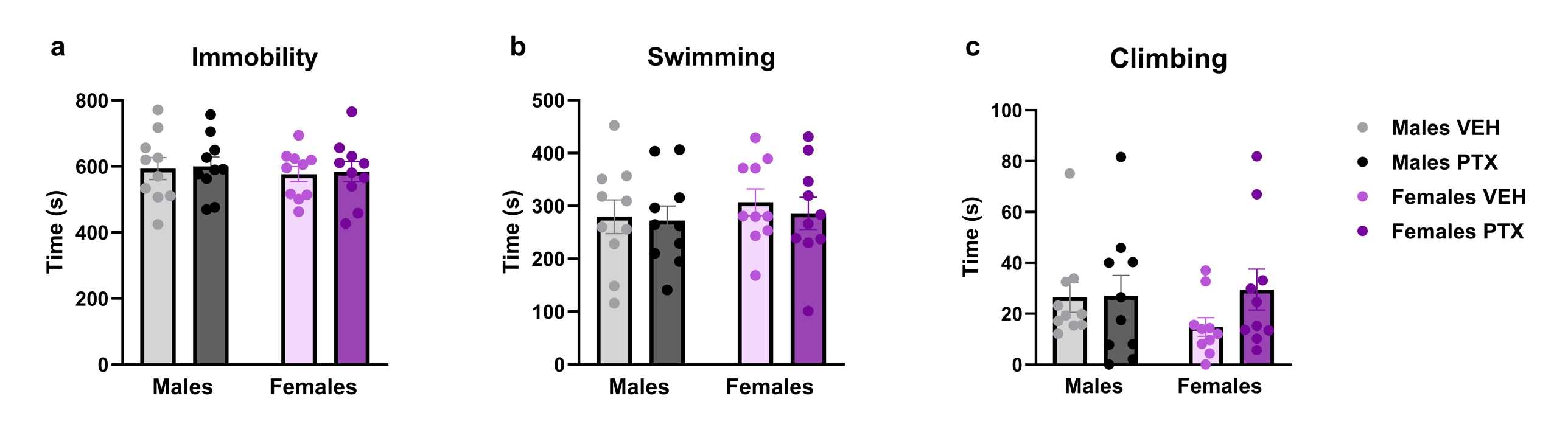
**

**Figure S3.** Forced Swim test. Time spent immobile (a), swimming (b), and climbing (c) during the pre-test phase. Data expressed as mean ± S.E.M (n=10 per group).
